# *Tinospora cordifolia*‐associated hepatotoxicity has been scientifically misconstrued, in haste

**DOI:** 10.1002/hep4.2050

**Published:** 2022-07-19

**Authors:** Acharya Balkrishna, Kunal Bhattacharya, Sandeep Sinha, Rishabh Dev, Jyotish Srivastava, Swati Haldar, Anurag Varshney

**Affiliations:** ^1^ Drug Discovery and Development Division Patanjali Research Institute Haridwar India; ^2^ Department of Allied and Applied Sciences University of Patanjali Haridwar India; ^3^ Department of Biology Patanjali Research Institute Haridwar India; ^4^ Department of Chemistry Patanjali Research Institute Haridwar India; ^5^ Department of Microbiology Patanjali Research Institute Haridwar India; ^6^ Special Centre for Systems Medicine Jawaharlal Nehru University New Delhi India

To the editor,

The well‐known medicinal herb *Tinospora cordifolia* is used in various classical Ayurvedic medicines. *T. cordifolia*‐induced hepatotoxicity, reported by Kulkarni et al.,^[^ ^1^ ^]^ has several lacunae, such as use of unauthenticated plant material(s), random dosage, unsupervised self‐medication, and preexisting comorbidities, that are likely to compromise the study outcomes. Interestingly, the median *T. cordifolia* consumption of 40 mL/day presented in the study is much higher than the permitted limit (https://tinyurl.com/5n6cn25u). Kulkarni et al.^[^ ^1^ ^]^ have attributed the apparent hepatotoxicity to plant alkaloids, terpenoids, and polysaccharides, without mentioning their quantities. Precise quantities and identities of phytocompounds are important for evidence‐based toxicity reporting of traditional herbal medicines. Five patients (11.6%) also intermittently consumed other herbal extracts independently, some with known hepatic cytochrome P450 modulatory activities. Moreover, independent acute toxicological studies have shown that up to 2000 mg/kg/day of *T. cordifolia* aqueous extract is safe in rats.^[^ ^2^ ^]^ Clerodane furano‐diterpenoids are isolated in chloroform extraction or through several steps of purification of aqueous alcoholic extract.^[^ ^3^ ^]^ Therefore, their role in observed hepatotoxicity, as inferred by Kulkarni et al., is unlikely. The association of *T. cordifolia* and its phytocompound arabinogalactane polysaccharides with B‐cell activation and concomitant development of autoimmune hepatitis is also misconstrued. Additionally, the phytochemicals identified in different *T. cordifolia* formulations by Kulkarni et al. do not match the reported signature phytocompounds of *T. cordifolia*.^[^ ^4^ ^]^ This could largely be due to choosing gas chromatography–tandem mass spectrometry (GC/MS–MS), not really the optimal tool for the intended purpose, over liquid chromatography/MS–MS or high‐performance liquid chromatography techniques for analysis. Besides, it is challenging to measure inorganic compounds using GC/MS–MS, as reported by Kulkarni et al. The heavy metal contaminations detected by Kulkarni et al. in most of the *T. cordifolia* formulations were within permitted limits as per the Ministry of Ayush, Government of India guidelines related to herbal preparations (https://tinyurl.com/2w4235rt) and the International Council for Harmonisation of Technical Requirements for Pharmaceuticals for Human Use guidelines for medicinal products (https://tinyurl.com/4skw2jvv). Nonetheless, there are no defined safety limits for dose and exposure to heavy metals from drugs or supplements, and ideally, herbal medicines should not contain heavy metals. Therefore, the current evidence presented by Kulkarni et al., as per our understanding, may not associate *T. cordifolia* with observed hepatotoxicity. Rather, this study restates that unsupervised self‐medication should be discouraged even for natural medicines, and public awareness in this regard should be raised.

## CONFLICTS OF INTEREST

Acharya Balkrishna is a trustee in Divya Yog Mandir Trust, Haridwar, India, which governs Divya Pharmacy, Haridwar; he is one of the founding promoters and holds an honorary managerial position in Patanjali Ayurved Ltd, Haridwar. Divya Pharmacy and Patanjali Ayurveda Ltd commercially manufacture and sell several ayurvedic products, some of which include *T. cordifolia* as an herbal component. Kunal Bhattacharya, Sandeep Sinha, Rishabh Dev, Jyotish Srivastava, Swati Haldar, and Anurag Varshney are employed by Patanjali Research Institute, which is governed by the Patanjali Research Foundation Trust, a not‐for‐profit organization.
